# Epigenome-wide analysis of aging effects on liver regeneration

**DOI:** 10.1186/s12915-023-01533-1

**Published:** 2023-02-13

**Authors:** Junying Wang, Wen Zhang, Xiaoqin Liu, Minjee Kim, Ke Zhang, Robert Y. L. Tsai

**Affiliations:** 1 Institute of Biosciences and Technology, Texas A&M Health Science Center, 2121 W. Holcombe Blvd, Houston, TX 77030 USA; 2 Department of Translational Medical Sciences, Texas A&M Health Science Center, 2121 W. Holcombe Blvd, Houston, TX 77030 USA

**Keywords:** DNA methylation, Epigenetic, Tissue repair, WGBS

## Abstract

**Background:**

Aging is known to exert an effect on liver regeneration, with the ability of liver to regenerate displaying a significant decline over time. Liver physiological parameters such as liver volume, blood flow, and metabolism, as well as the ability to regenerate after injury have all been shown to decrease at old age in humans and model systems, with a number of molecular mechanisms proposed to be involved, including DNA methylation-dependent genome remodeling. To address how changes in DNA methylation mediate the adverse aging effect on liver regeneration, we searched for differentially methylated genomic regions (DMRs) in mouse livers co-regulated by aging and regeneration and determined their associated genes and enriched pathways.

**Results:**

DMRs were identified using whole-genome bisulfite sequencing (WGBS). Pathway analysis of aging DMR-mapped genes revealed two distinct phases of aging, 2-to-8 and 8-to-16 months old (m/o). Regenerative DMR-mapped differentially expressed genes (DEGs) were enriched in pathways controlling cell proliferation and differentiation. Most DMRs shared by both aging and regeneration changed in the same methylation direction between 2 and 8 m/o but in the opposite direction between 8 and 16 m/o. Regenerative DMRs inversely affected by aging during 8-to-16 m/o were found in the promoter/gene regions of 12 genes. Four regenerative DEGs were synchronously regulated by early aging and inversely regulated by mid-to-late aging DMRs. Lead DMR-mapped genes were validated by their expression profiles in liver aging and regeneration.

**Conclusions:**

Our study has uncovered new DMRs and gene targets inversely affected by liver aging and regeneration to explain the adverse aging effect on liver regeneration. These findings will be of fundamental importance to understand the epigenomic changes underlying the biology of aging on liver regeneration.

**Supplementary Information:**

The online version contains supplementary material available at 10.1186/s12915-023-01533-1.

## Background

Liver is known to undergo significant changes at old age [1–10]. Compared to those less than 40 years old (y/o), people more than 65 y/o show decreased liver volume by 20–40%, reduced blood flow by 35%, decreased metabolism of LDL cholesterol by 35%, and elevated serum γ-glutamyltransferase and alkaline phosphatase levels [9]. The ability of liver to regenerate after surgery or injury is also compromised in old age. Hepatocytes that enter the S-phase after partial hepatectomy (PHx) are less in aged (70%) compared to young livers (99%), and those that replicate do so less rapidly [2]. Consequently, the restored liver mass after damage is reduced and delayed in the elderly, increasing the mortality and morbidity of liver diseases [11–16].

Several mechanisms have been raised to explain the adverse aging effect on liver regeneration, including a decreased hepatic sensitivity to growth factors [17, 18], increased reactive oxygen species by p66^shc^ expression/phosphorylation [19], shortened telomere length [20, 21], and reduced pseudocapillarization. At the transcriptional level, aging increases the activities of C/EBP and E2F4-Rb to diminish the expression of hepatocyte proliferative factors [5, 6, 22–25]. Epigenetically, it was shown that C/EBPα interacts with Brm to inhibit the expression of c-Myc, b-Myb, cdc2, FoxM1B, and E2F target genes [6, 22, 23, 26] or interacts with HDAC1 to inhibit the expression of c-Myc and FOXM1B [22]. The complexing of C/EBPα with Brm or HDAC1 is regulated by phosphorylation on C/EBPα-S193, which is increased by age via a cyclin D3 and GSK3β-mediated mechanism [27]. Aging also increases the translation of C/EBPβ and the level of the C/EBPβ-HDAC1 complex, which represses GSK3β and SIRT1 promoters, causing impaired body homeostasis and liver proliferation [8, 28].

In humans, age-dependent DNA methylation remodeling of the genome has been proposed as a driving mechanism of many age-related events, occurring before adulthood and continuing through 60 years of age [29–31]. Genome-wide DNA methylation studies on liver aging were mostly conducted using the BeadChip technology, which covers up to 850 K CpG sites in humans (~ 3% of the total 28 M sites) or 350 K-1.5 M CpG sites in mice (~ 0.01% to 6.8% of the total 21.3 M sites) [30, 32–35]. Two studies that used the whole-genome bisulfite sequencing (WGBS) approach investigated the dietary effect on DNA methylation during liver aging [36, 37]. To date, no WGBS study on DNA methylation in liver regeneration has been reported [38].

In this study, we applied WGBS to identify differentially methylated regions (DMRs) in the whole genome during the aging and regeneration of mouse livers and determined how aging and regenerative DMRs relate to each other and with differentially expressed genes (DEGs) induced by liver regeneration. Here, the term “aging” refers to biological changes that happen with time across the whole lifespan, including developmental milestones in childhood and adolescence, whereas the terms “aged” or “senescence” refers to functional impairments that commonly occur at old age. C57BL/6 mice show three maturation phases in life, including mature adult (3–6 months old), middle-aged (10–14 months old), and old (18–24 months old), which correspond to 20–30, 38–47, and 56–69 years old in humans, respectively. The age range for mouse life phases is characterized based on a cohort of 150 males and 150 females. To identify the DMR events leading into each of the three life phases, thereby maximizing the chance of finding driver events instead of adaptive or phenotypic events, we chose three age groups (2, 8, and 16 m/o) for aging analysis and referred to 8-to-2-m/o and 16-to-8-m/o changes as early and mid-to-late aging, respectively. Based on the literature, liver regeneration has already shown significant decrease in 12-m/o mice [24] and 12–15-m/o rats (1) compared to young mice (2 m/o) and rats (4 w/o and 4 m/o), respectively. To identify healthy regeneration-induced DMRs, we used 2-m/o livers at 1 day (1d), 2d, and 4d following 70% partial hepatectomy (PHx). WGBS was used to provide an unbiased whole-genome single nucleotide approach that allowed maximal discovery of DMRs synchronously or inversely cross-regulated by liver regeneration and aging. Our results revealed novel DMR-mapped gene targets underlying the age-dependent decline in liver regeneration.

## Results

### Whole-genome analysis of liver aging DMRs from 2-to-8-to-16 m/o

**Fig. 1:**
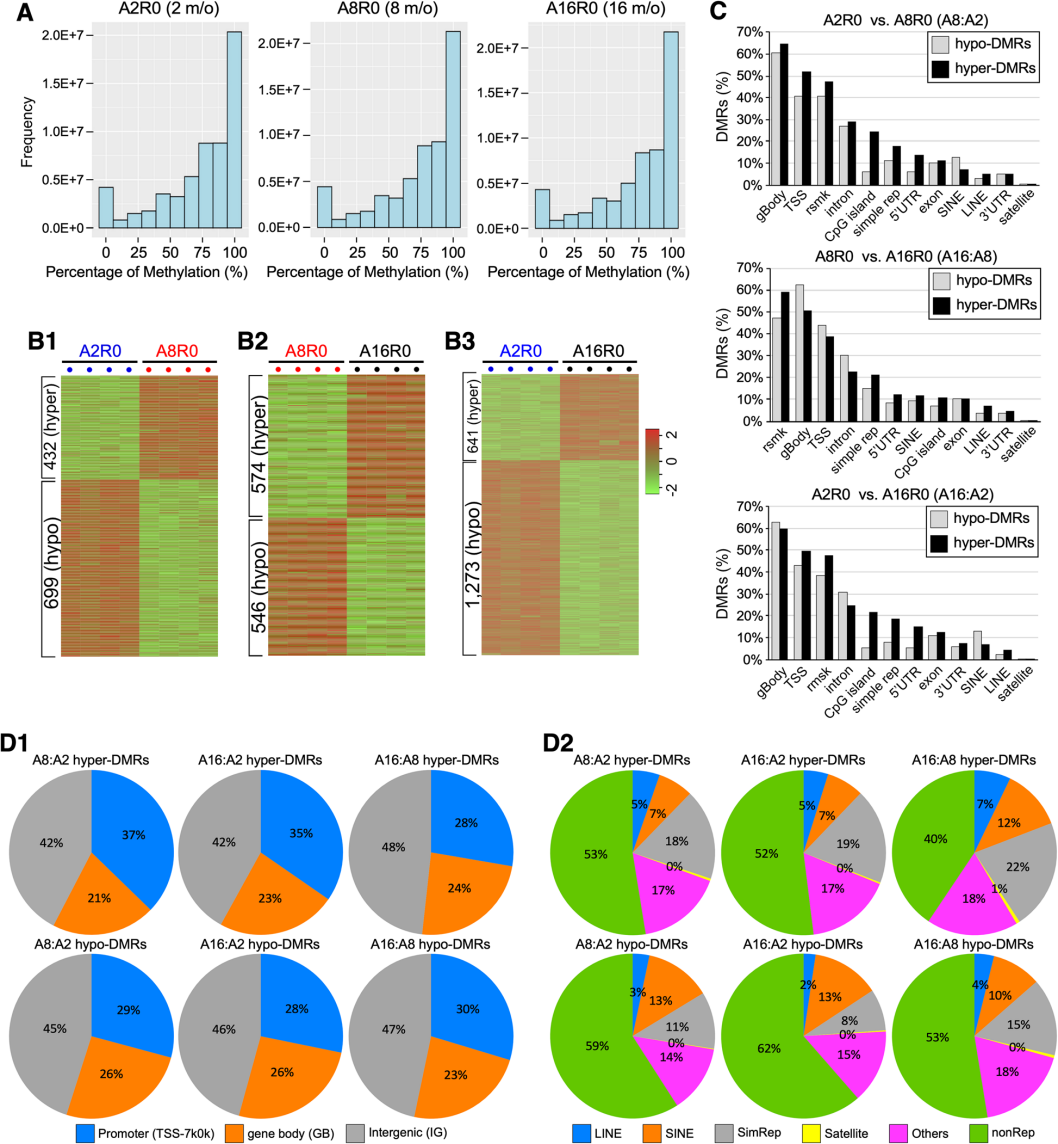
Discovery and characterization of differentially methylated regions (DMRs) in aging livers by whole-genome bisulfite sequencing (WGBS). **A** Histograms of CpG event frequencies categorized by their DNA methylation levels for three baseline age groups (A2R0, A8R0, and A16R0), where the *X*-axis shows the DNA methylation level (from 0 to 100%) and the *Y*-axis shows the CpG event frequency. Each graph represents the average of four biological samples. **B** Heat maps of aging DMRs by comparing 8-m/o (A8R0) to 2-m/o (A2R0) livers (**B1**), 16-m/o (A16R0) to A8R0 livers (**B2**), and A16R0 to A2R0 livers (**B3**). Color scale indicates *Z* score, showing increased or decreased methylation by red or green color, respectively. Hyper-DMRs are listed on top of hypo-DMRs. R0 indicates the non-regenerative condition. **C** Genomic distributions of hypo-DMRs (*grey bars*) and hyper-DMRs (*black bars*) in comparing A8R0-to-A2R0, A16R0-to-A8R0, or A16R0-to-A2R0 livers. Abbreviations: rsmk, repetitive DNA regions; gBody, gene body; TSS, transcription start site; rep, repeat; SINE, short interspersed nuclear elements; LINE, long interspersed nuclear elements; UTR, untranslated region. *Y*-axis indicates the percentage (%) of total hypo-DMRs or hyper-DMRs found in specific genomic regions of interest (hotspot analysis, see the “Methods” section). Different genomic regions are not mutually exclusive. **D** Pie charts of aging DMR percentages found in genomic regions containing gene structures versus intergenic regions (**D1**) or in repeat versus non-repeat sequences (**D2**). Abbreviations: TSS-7 k, within 7kB upstream of TSS; GB, gene body; IG, intergenic region; LINE, long interspersed nuclear elements; SINE, short interspersed nuclear elements; SimRep: simple repeats (micro-satellites); Satellite, satellite repeats; Others: other repeat categories as defined in RMSK from the UCSC genome browser (https://genome.ucsc.edu/cgi-bin/hgTables?db=hg38&hgta_group=rep&hgta_track=rmsk&hgta_table=rmsk&hgta_doSchema=describe+table+schema); nonRep: non-repeat regions

Whole-genome DNA methylation changes occurring between 2-m/o (A2), 8-m/o (A8), and/or 16-m/o (A16) mouse livers were determined by WGBS (Additional file 1: Fig. S1A). Bioanalyzer results showed that average insert sizes for bisulfite converted libraries were ~ 320 bp (Additional file 1: Fig. S1B). The sequenced data showed an average tenfold coverage of the whole mouse epigenome, where > 17 million CpGs (> 80% of the total 21.3 million CpGs) had at least threefold coverage (Additional file 1: Fig. S1C). Each liver sample is a pool of millions of cells. While DNA methylation on a single CpG site is binary (0 or 1) in nature at the single cell level, the average methylation of such a site from a pool of cells represents a gradient from 0 to 100%. WGBS data were analyzed using the MOABS software [39] that calculated the average methylation percentages across all CpG sites. Raw DNA methylation data of all samples were deposited individually in the GEO database (GSE211999). The levels of DNA methylation for each age group (representing the average of four biological samples) were displayed in histograms, where the *X*-axis showed the DNA methylation level (from 0 to 100%), and the *Y*-axis showed the event frequency (Fig. 1A). A DMC is defined by a change in the average methylation percentage of a CpG site whose absolute value is > 0.2 (20%) in one sample compared to another. A DMR is defined by ≥ 3 consecutive DMCs within 300 bp with the step size of 1 nt. 105,264 differentially hyper-methylated CpGs (hyper-DMCs) and 90,280 hypo-DMCs were identified by comparing 8-m/o to 2-m/o livers (A8:A2), which corresponded to 432 hyper-DMRs and 699 hypo-DMRs (Fig. 1B1, Additional file 3: Table 1). 98,810 hyper-DMCs (574 hyper-DMRs) and 93,115 hypo-DMCs (546 hypo-DMRs) were identified by comparing 16-m/o to 8-m/o livers (A16:A8) (Fig. 1B2, Additional file 3: Table 1). 119,899 hyper-DMCs (641 hyper-DMRs) and 102,384 hypo-DMCs (1,273 hypo-DMRs) were identified by comparing 16-m/o to 2-m/o livers (A16:A2) (Fig. 1B3, Additional file 3: Table 1).

**Fig. 2:**
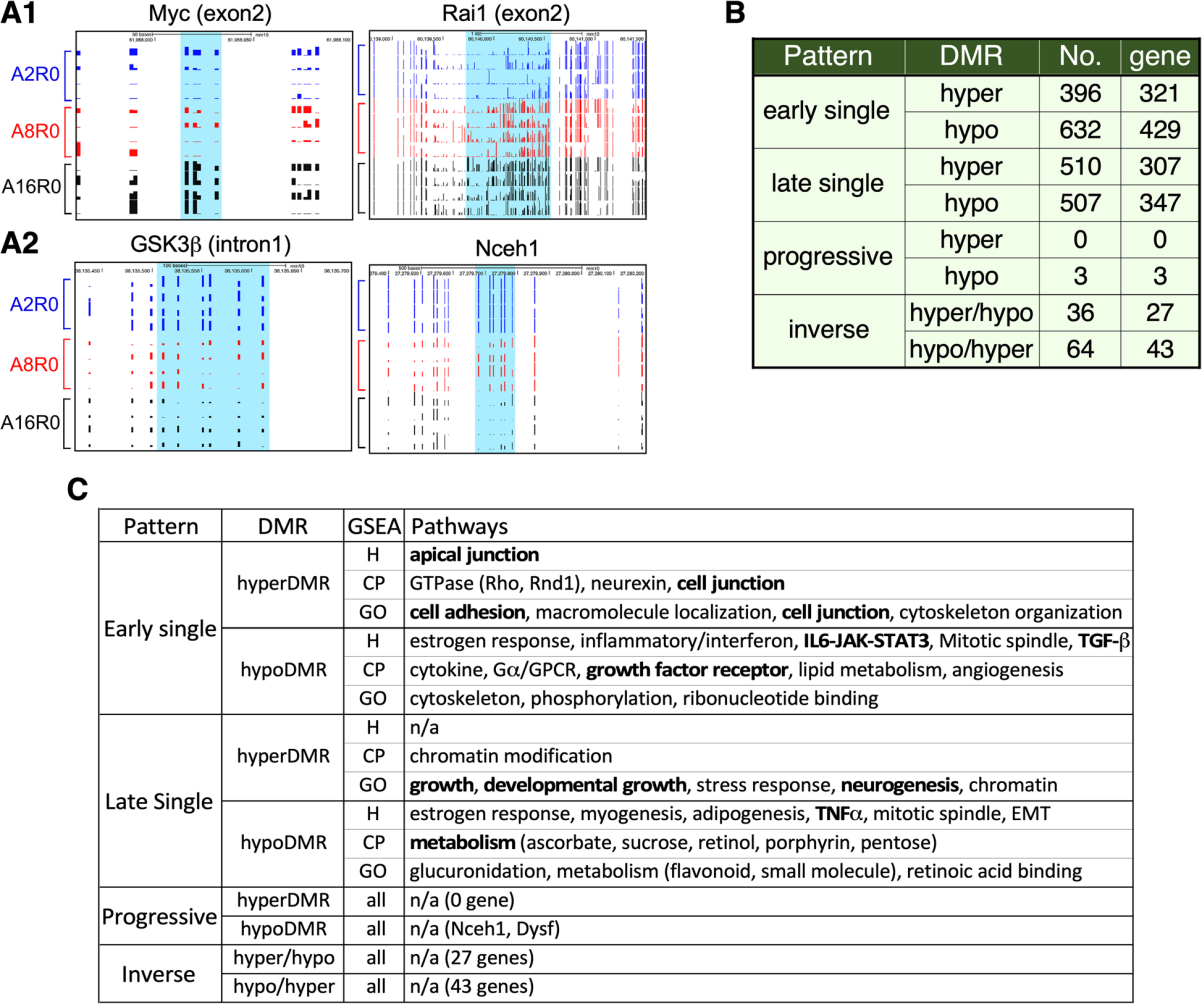
Examples and pathway analysis of aging liver DMRs. **A** Genome-browser (TRACK) views displaying two aging hyper-DMRs associated with Myc and Rai1 (**A1**) and two hypo-DMRs associated with GSK3β and Nceh1 (**A2**). DMRs are highlighted in blue shade. **B** Numbers of DMRs (No.) and their mapped genes in the early single, late single, progressive, and inverse categories. **C** GSEA analyses of genes mapped by the four different types of DMRs using the Hallmark (H), Canonical Pathway (CP), and Gene Ontology (GO) filters

High percentages of the aging DMRs were found in two types of genomic regions (Fig. 1C, D, and Additional file 2: Fig. S2). One is related to gene structures, such as the transcription start site (TSS), gene body (gBody), 5′-untranslated regions (UTR), and 3′-UTR, indicating their potential roles in gene expression regulation. The other is related to repetitive elements listed in the rmsk (RepeatMaster track), including short interspersed nuclear elements (SINEs), long interspersed nuclear elements (LINEs), simple (microsatellite) or satellite repeats, and others, indicating their roles in genomic stability maintenance. gBody, TSS, and repeat sequences were the three most common genomic regions where aging DMRs were found. On average, 55% (52–58%) of the aging DMRs were associated with gene structures (Fig. 1D1 and Additional file 2: Fig. S2A), and 47% (39–59%) were associated with repeat sequences (Fig. 1D2 and Additional file 2: Fig. S2B). The percentages of hyper-DMRs on CpG islands are significantly higher than that of hypo-DMRs in 8 or 16 m/o compared to 2 m/o. Two examples of aging hyper-DMRs were found in association with c-Myc and Rai1 (Fig. 2A1). Aging increased DNA methylation in the gBody (exon2) region of c-Myc (chr15:61,988,015–61,988,032) between 2 and 16 m/o and in the gBody (exon2) region of Rai1 (chr11:60,140,252–60,140,367) between 2 and 8 m/o. c-Myc is known to be activated during liver regeneration and suppressed by C/EBPα. Rai1 has not yet been implicated in liver aging or regeneration. Two examples of aging hypo-DMRs were found in association with GSK3β and Nceh1 (Fig. 2A2). Aging decreased DNA methylation in the gBody (intron1) region of GSK3β (chr16:38,135,334–38,135,558) between 2 and 8 m/o and in the gBody region (ENSMUSG00000027698) of Nceh1 (chr3:27,279,398–27,279,785) in a progressive fashion from 2-to-8 to 16 m/o. Decreased expression of GSK3β has been associated with liver aging [27]. Nceh1 has not yet been implicated in liver aging or regeneration.

To dissect the biological events associated with the DNA methylation changes from 2-to-8 and from 8-to-16 m/o, we determined the numbers, genomic sites, and associated genes of aging DMRs that occurred between only 2 and 8 m/o (early single), only 8 and 16 m/o (late single), or both 2 and 8 and 8 and 16 m/o in a progressive or inverse fasion (Fig. 2B, Additional file 4: Table 2). Our results showed that most DMRs occurred as single events between 2 and 8 m/o (396 hyper-DMRs and 632 hypo-DMRs) or 8 and 16 m/o (510 hyper-DMRs and 507 hypo-DMRs). Only three DMRs showed progressive changes from 2-to-8 to 16 m/o. A sizable number of DMRs occurred in both 2-to-8 and 8-to-16 m/o but in an inverse fashion (36 hyper/hypo-DMRs and 64 hypo/hyper-DMRs). These findings indicated that early and mid-to-late aging DMRs are associated with genes of distinct biological functions. In the event that they are associated with the same genes, early and mid-to-late aging DMRs are often inversely methylated. Mapping of DMRs to both TSS and gBody regions identified 750 genes in the early single group, 654 genes in the late single group, 3 genes in the progressive group, and 70 genes in the inverse group. Gene Set Enrichment Analysis (GSEA) analyses revealed pathways enriched in genes mapped by early and late single DMRs (Fig. 2C). Notably, genes mapped by early single hyper-DMRs were enriched in the cell adhesion pathway, whereas genes mapped by early single hypo-DMRs were enriched in the Jak-STAT, TGF-beta, and growth factor receptor pathways. Contrarily, genes mapped by late single hyper-DMRs were enriched in the growth, develpmental growth, and chromatin modification pathways, whereas genes mapped by late single hypo-DMRs were enriched in the TNF-alpha and metabolic pathways (Fig. 2C, bolded). No specific pathways were found enriched by the progressive or inverse DMRs due to their low numbers. These results show that early (2-to-8-m/o) and mid-to-late (8-to-16-m/o) aging DMRs are indicative of downstream events of growth/regeneration and aging/degeneration, respectively.

### Discovery of whole-genome DMRs during liver regeneration

**Fig. 3:**
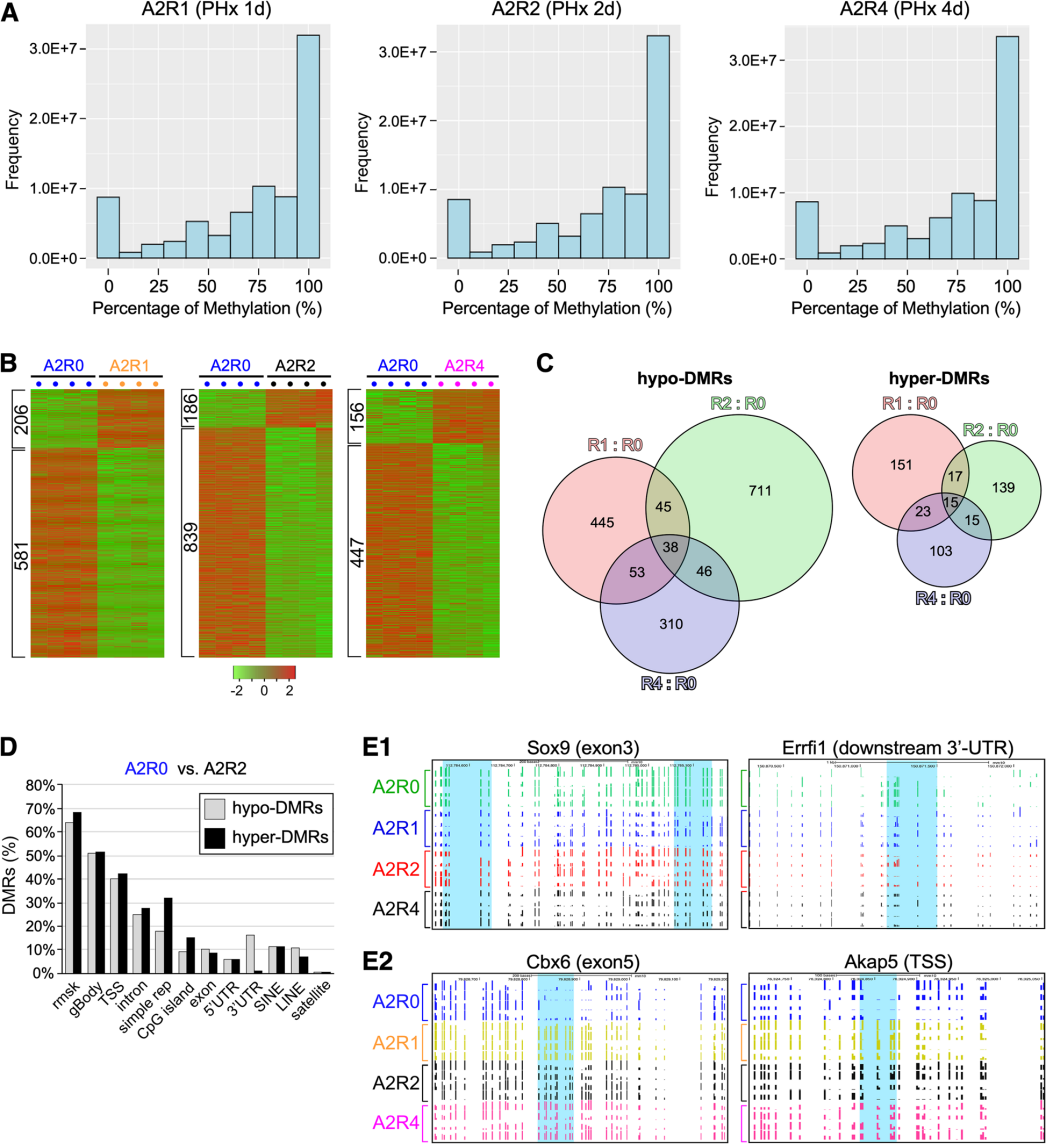
Discovery and characterization of DMRs in regenerating livers by WGBS. **A** Histograms of CpG event frequencies categorized by their DNA methylation levels for three 2-m/o PHx groups (A2R1, A2R2, and A2R4), where the *X*-axis shows the DNA methylation level (from 0 to 100%) and the *Y*-axis shows the CpG event frequency. Each graph represents the average of four biological samples. **B** Heat maps of regenerative DMRs found in regenerating 2-m/o livers collected on 1d (A2R1), 2d (A2R2), and 4d (A2R4) after 70% partial hepatectomy (PHx) compared to baseline livers collected before the surgery (A2R0). Hyper-DMRs are listed on top of hypo-DMRs. **C** Venn diagrams of unique and overlapped hypo-DMRs and hyper-DMRs shared by the R1:R0 (red), R2:R0 (green), and/or R4:R0 (blue) groups. **D** Genomic distributions of regenerative DMRs by comparing A2R2 to A2R0 liver samples. **E** Genome-browser (TRACK) views displaying two regenerative hypo-DMRs associated with Sox9 and Errfi1 (**E1**) and two hyper-DMRs associated with Cbx6 and Akap5 (**E2**)

Regenerative DMRs were determined by measuring DNA methylation levels of 2-m/o (A2) liver samples at 1d (A2R1), 2d (A2R2), and 4d (A2R4) post-PHx and comparing them to non-regenerative samples collected before the surgery (A2R0). Analyses of the WGBS data showed bisulfite conversion rates > 99% and an average tenfold coverage of the whole epigenome (Additional file 1: Fig. S1D). Raw DNA methylation data of all samples were deposited in the GEO database (GSE211999). The levels of DNA methylation for each PHx group (representing the average of four biological samples) were displayed in histograms (Fig. 3A). Compared to the baseline non-regenerative samples (A2R0), we identified 206/581 hyper/hypo-DMRs in 1d post-PHx livers (A2R1), 186/839 hyper/hypo-DMRs in 2d livers (A2R2), and 156/447 hyper/hypo-DMRs in 4d livers (A2R4) (Fig. 3B, Additional file 5: Table 3). Among those DMRs, there are 32/83 hyper/hypo-DMRs shared by the R1:R0 and R2:R0 groups, 38/91 hyper/hypo-DMRs shared by the R4:R0 and R1:R0 groups, 30/83 hyper/hypo-DMRs shared by the R4:R0 and R2:R0 groups, and 15/38 hyper/hypo-DMRs shared by all three (Fig. 3C, Additional file 6: Table 4). Hostspot analyses showed that the majority of regenerative DMRs (R2:R0) were found in genomic regions associated with gene structures (e.g., TSS and gBody) or repeat sequences (e.g., rsmk) (Fig. 3D). Two examples of regenerative hypo-DMRs were found in association with Sox9 and Errfi1. Regeneration decreased DNA methylation in the gBody (exon3) region of Sox9 (chr11:112,784,975–112,785,325) and in a region (chr4:150,871,175–150,871,499) located 2.3 kb downstream of the 3′-UTR of Errfi1 in liver samples collected at 1d, 2d, and 4d post-PHx compared to pre-PHx samples (Fig. 3E1). In consistence, Sox9 is up-regulated during liver regeneration [40, 41]. The role of Errfi1, also known as mitogen-induced gene 6 (mig-6), in liver regeneration is unclear but has been reported to be down-regualted in hepatocellular carcinoma [42]. Two examples of regenerative hyper-DMRs were found in association with Chrombox 6 (Cbx6) and A-kinase anchoring protein 5 (Akap5) (Fig. 3E2). Regeneration increased DNA methylation in the gBody (exon5) region of Cbx6 (chr15:79,828,706–79,828,923) and in the TSS (− 39 to − 17) region of Akap5 (chr12:76,324,852–76,324,874) at 1d, 2d, and 4d compared to 0d. Cbx6 is a transcription repressor, whose overexpression was shown to contribute to the progression of hepatocellular carcinoma, thus predicting a poor prognosis [43]. Akap5 was shown to regulate the activity of the vascular L-type Ca^2+^ channel in response to elevated glucose [44], but its function in liver regeneration remains unclear.

### Mapping regenerative DMRs to the promoter regions of differentially expressed genes in regeneration

**Fig. 4:**
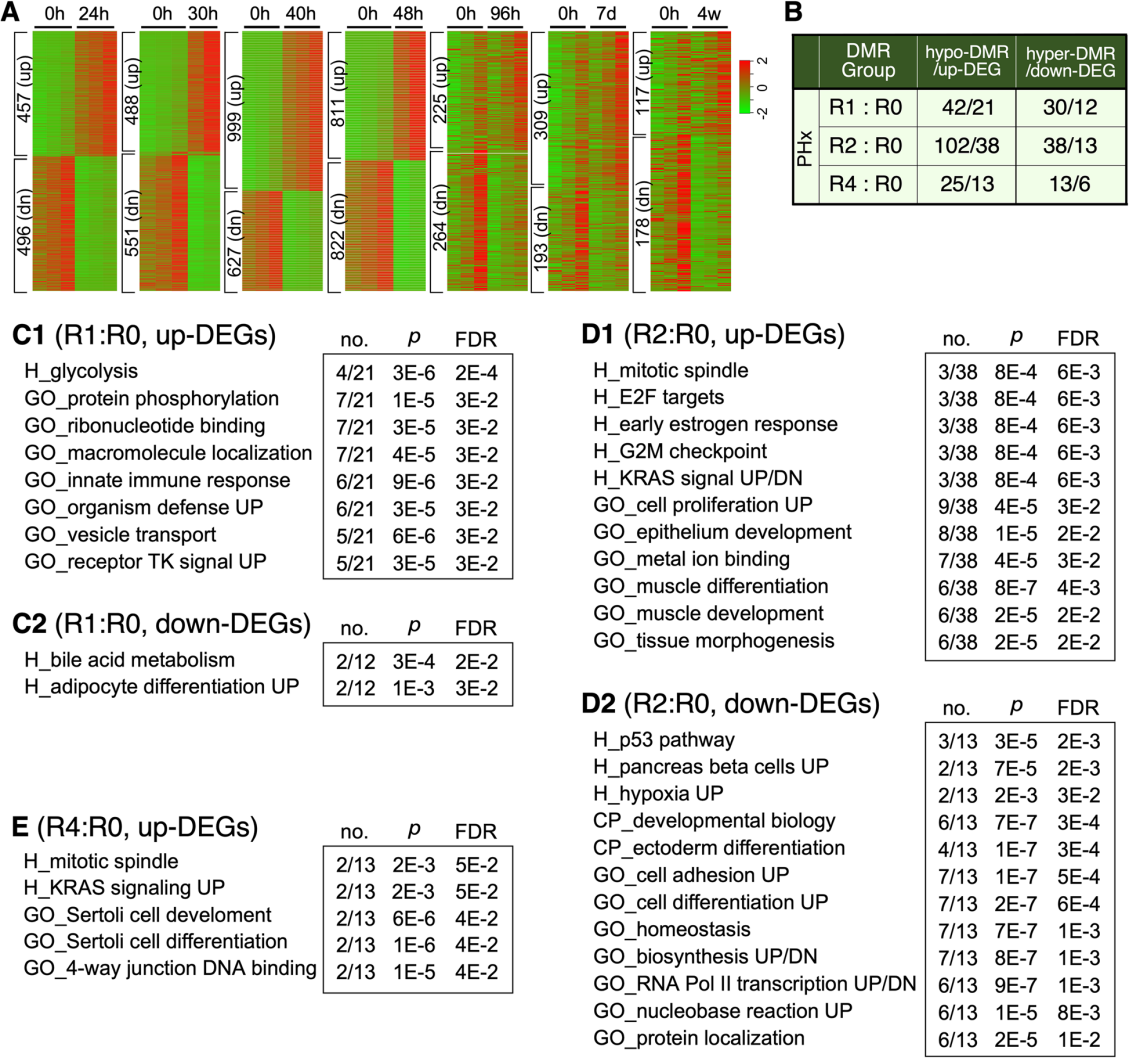
Mapping of regenerative DMRs to differentially expressed genes (DEGs) during liver regeneration. **A** Heat maps of DEGs at different time points of liver regeneration after PHx. Numbers of up- (top, up) and down-regulated genes (bottom, dn) are indicated on the left. Hour (h), day (d), week (w). **B** Numbers of up-regulated DEGs (up-DEGs) mapped by regenerative hypo-DMRs and down-regulated DEGs (down-DEGs) mapped by regenerative hyper-DMRs in the R1:R0, R2:R0, and R4:R0 groups. Gene Set Enrichment Analysis (GSEA) showing pathways enriched in up-DEGs and down-DEGs mapped by regenerative hypo-DMRs and hyper-DMRs, respectively, in the R1:R0 (**C**), R2:R0 (**D**), and R4:R0 (**E**) paradigms. Abbreviations: H, hallmark; CP, canonical pathway; GO, gene ontology; FDR, false discovery rate; n1/n2, the number of DEGs enriched in specific pathways (n1) over the total number of query DEGs (excluding putative genes) (n2)

To identify regenerative DMRs with functional roles in regulating gene expression in response to PHx-induced liver regeneration, raw RNA-seq data were extracted from a GEO dataset (GSE125007) containing transcriptomic data of 8–12-w/o mouse livers before and after partial hepatectomy at 24 h (h), 30 h, 40 h, 48 h, 96 h, 7 days (d), and 4 weeks (w) [45]. RNA-seq data were analyzed and mapped to the mouse genome. Uniquely mapped reads were used to assemble the transcriptome and quantify gene expression levels with reads per kb per million (FPKM). DEGs were identified as those with corrected *p* values ≤ 0.05 and absolute fold changes ≥ 2. The numbers of up-regulated DEGs (up-DEGs) and down-regulated DEGs (down-DEGs) were within the range of 457–999 for the first 48 h post-PHx and began to drop after 96 h to 117–309 (Fig. 4A). We then mapped regenerative hypo-DMRs to the promoter regions (− 7 to + 3 kB of the TSS) of up-DEGs and hyper-DMRs to the promoter regions of down-DEGs. Forty-two hypo-DMRs (21 up-DEGs) and 30 hyper-DMRs (12 down-DEGs) were mapped in the R1:R0 paradigm. One hundred two hypo-DMRs (38 up-DEGs) and 38 hyper-DMRs (13 down-DEGs) were found in the R2:R0 paradigm. Twenty-five hypo-DMRs (13 up-DEGs) and 13 hyper-DMRs (6 down-DEGs) were found in the R4:R0 paradigm (Fig. 4B, Additional file 7: Table 5).

**Fig. 5:**
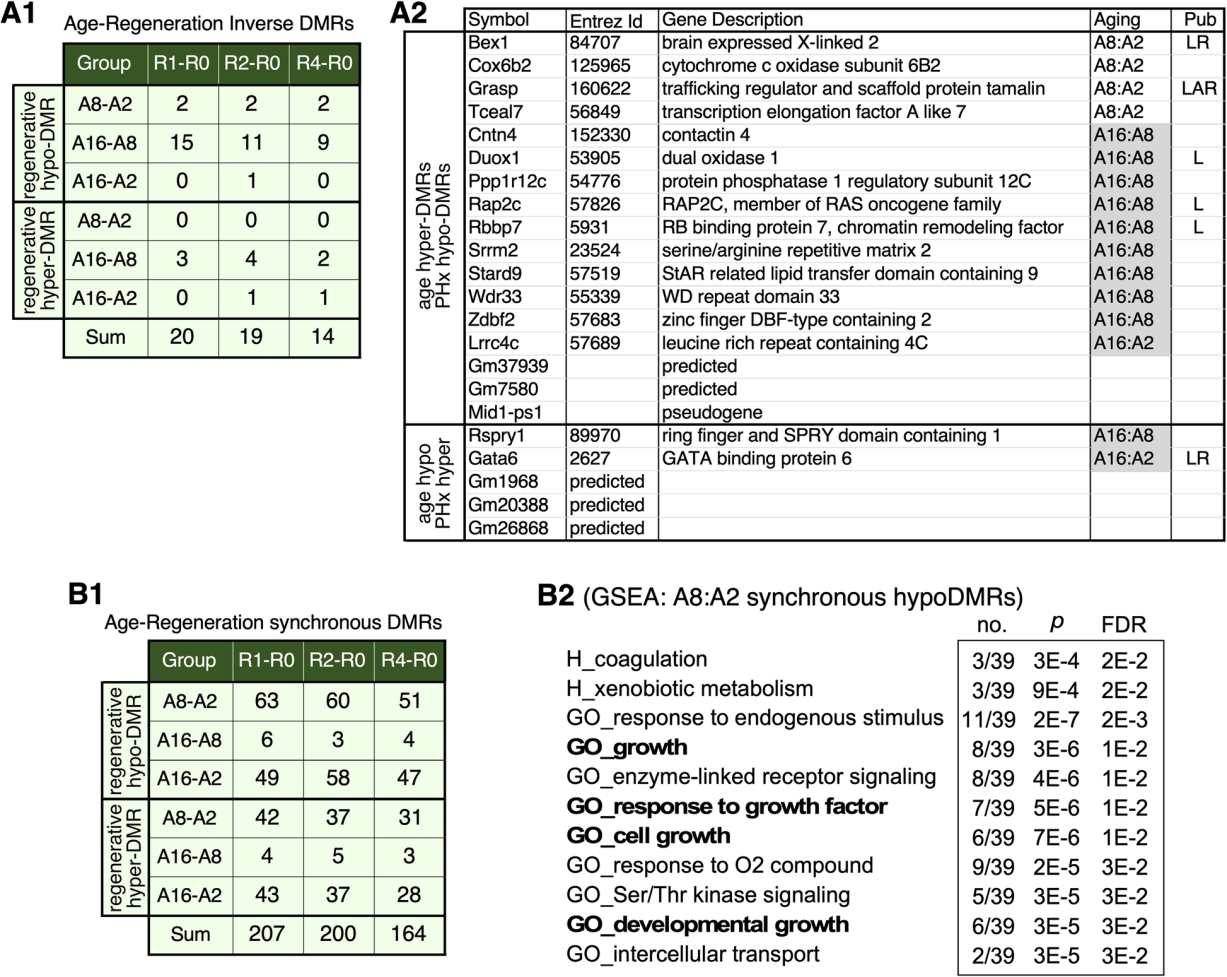
Synchronous and inverse changes between aging and regenerative DMRs. (**A1**) Numbers of regenerative hypo-DMRs hypermethylated during aging (top) and hyper-DMRs hypomethylated during aging (bottom). (**A2**) Lists of genes mapped by age-inverse regenerative hypo-DMRs (upper panel) or hyper-DMRs (lower panel), their aging paradigms, and liver-related publications found by PubMed search. Six showed liver-related functions (L), with or without additional functions in regeneration (R) or aging (A). **B1** Numbers of DMRs that are synchronously hypomethylated or hypermethylated during liver aging and regeneration. **B2** Lists of Hallmark (H) and Gene Ontology (GO) pathways enriched in genes whose promoter regions were mapped by early (A8:A2) age-synchronous regenerative hypo-DMRs

Pathways enriched in the regenerative DMR-mapped DEGs were analyzed by GSEA using the Hallmark (H), Canonical Pathway (CP), and Gene Ontology (GO) gene set filters with FDR *q*-value cut-off set at < 0.05. All Hallmark-identified pathways, CP-identified pathways with ≥ 4 enriched genes, and GO-identified pathways with ≥ 5 enriched genes were listed (Fig. 4C–E). Pathways enriched in DMR-mapped DEGs were found more in the R2:R0 paradigm than in the R1:R0 or R4:R0 paradigm. Pathways enriched in R1:R0 hypo-DMR-mapped up-DEGs include those involved in immediate early responses (e.g., protein phosphorylation, receptor tyrosine kinase signaling, and immune), glycolysis, and ribonucleotide binding (Fig. 4C1), whereas pathways enriched in R1:R0 hyper-DMR-mapped down-DEGs include those involved in bile acid metabolism and adipogenesis (Fig. 4C2). Pathways enriched in hypo-DMR-mapped up-DEGs in the R2:R0 paradigm include those involved in cell proliferation, cell cycle regulation, tissue development/differentiation, early estrogen response, and KRAS signaling (Fig. 4D1), whereas pathways enriched in hyper-DMR-mapped down-DEGs in the R2:R0 paradigm include those involved in p53 response, hypoxia response, cell adhesion, and several other cell biological events (Fig. 4D2). There were only a few pathways enriched in hypo-DMR-mapped up-DEGs in the R4:R0 group, including those involved in mitotic spindle, KRAS UP, and Sertoli cell development/differentiation (Fig. 4E). No enriched pathway was found for down-DEGs mapped by the R4:R0 hyper-DMRs.

### Analysis of regenerative DMRs showing age-dependent changes

To identify potential targets on which aging might regulate liver regeneration via the DNA methylation mechanism, we determined regions showing DNA methylation changes during liver aging and regeneration either in the same methylation (synchronous) direction (hyper/hyper and hypo/hypo) or in the inverse direction (hyper/hypo and hypo/hyper). Our primary focus was on the age-inverse regenerative DMRs, which might underlie the negative effect of aging on liver regeneration. We identified only 42 regenerative hypo-DMRs that were hypermethylated by aging and 11 regenerative hyper-DMRs that were hypomethylated during aging (Fig. 5A1, Additional file 8: Table 6). Those age-inverse regenerative DMRs were found predominantly in the A16:A8 group (*n* = 44) compared to the A8:A2 group (*n* = 6) but showed no preferential distribution among different regeneration groups (R1:R0, R2:R0, and R4:R0). This finding suggests that mid-to-late aging and regeneration may represent biologically opposite events. GREAT analyses of age-inverse regenerative DMRs found no enriched GO term. We then mapped those DMRs to the TSS (-7 k + 3 k) and gene regions and identified 22 genes associated with age-inverse regenerative DMRs (Additional file 9: Table 7). Seventeen genes were mapped by age-inverse regenerative hypo-DMRs, including 14 known genes, two predicted genes, and one pseudogene. Among the 10 known genes mapped by the mid-to-late aging-inverse regenerative DMRs, three genes are related to liver pathophysiology, including the epigenetic silencing of Duox1 by promoter hypermethylation in hepatocellular carcinoma (HCC) [46], Rap2c-mediated inactivation of pathological fibrosis [47], and the unique expression of a spliced variant of Rbbp7 in HCC [48] (Fig. 5A2, top). Five genes were mapped by the age-inverse regenerative hyper-DMRs, including Rspry1 (ring finger and SPRY domain containing 1), Gata6, and three predicted genes (Fig. 5A2, bottom). Gata6 was connected to liver regeneration. GSEA found no pathway enriched in those genes mapped by age-inverse regenerative hyper- or hypo-DMRs due to their low numbers.

The numbers of DMRs that were synchronously hypomethylated (*n* = 341) or hypermethylated (*n* = 230) during liver aging and regeneration were significantly higher compared to those changed inversely (Additional file 8: Table 6). Those age-synchronous regenerative DMRs were found predominantly in the A8:A2 (*n* = 284) or A16:A2 (*n* = 262) group compared to the A16:A8 paradigm (*n* = 25), suggesting that early aging and regeneration may represent biologically similar events but showed no preferential distribution among different regeneration groups (Fig. 5B1). GREAT analyses showed that age-synchronous regenerative hypo-DMRs were enriched in 2 GO terms of Biological Process (sex differentiation and smooth muscle cell differentiation), 3 GO terms of Molecular Function (RNA polymerase II repressing transcription factor binding, repressing transcription factor binding, and RNA polymerase II transcription factor binding), 2 GO terms of Mouse Phenotype (early eyelid opening and abnormal mucous gland morphology), and 32 GO terms of Human Phenotype. No GO term was found for age-synchronous regenerative hyper-DMRs. Regenerative hypo-DMRs and hyper-DMRs showing synchronous changes during early aging (2-to-8 m/o) were mapped to the promoter regions (− 7 to + 3 kB, TSS-7 k + 3 k) of 61 and 28 genes, respectively (Additional file 9: Table 7). Thirty-nine of the 61 hypo-DMR-associated genes and 18 of the 28 hyper-DMR-associated genes were analyzed by GSEA (Hallmark, CP, and GO). Eleven pathways were enriched in genes mapped by A8:A2 age-synchronous regenerative hypo-DMRs, four of which were growth-related (growth, response to growth, cell growth, and developmental growth) (Fig. 5B2). Thirteen genes were enriched in two or more of the 11 pathways, including Gata4, Sox9, Usp9x (ubiquitin specific peptidase 9 X-linked), Lgmn (legumain), Epb41l5 (erythrocyte membrane protein band 4.1 like 5), Gnas, Rbbp7 (RB binding protein 7), Grb10 (growth factor receptor bound protein 10), Peg10 (paternally expressed 10), Gjb2 (gap junction protein beta 2), Tat (tyrosine aminotransferase), Hnf4A (hepatocyte nuclear factor 4 alpha), and TF (transferrin). No pathway was found enriched in genes mapped by age-synchronous regenerative hyper-DMRs.

### Age-dependent methylation on the promoter regions of regenerative DEGs

**Fig. 6:**
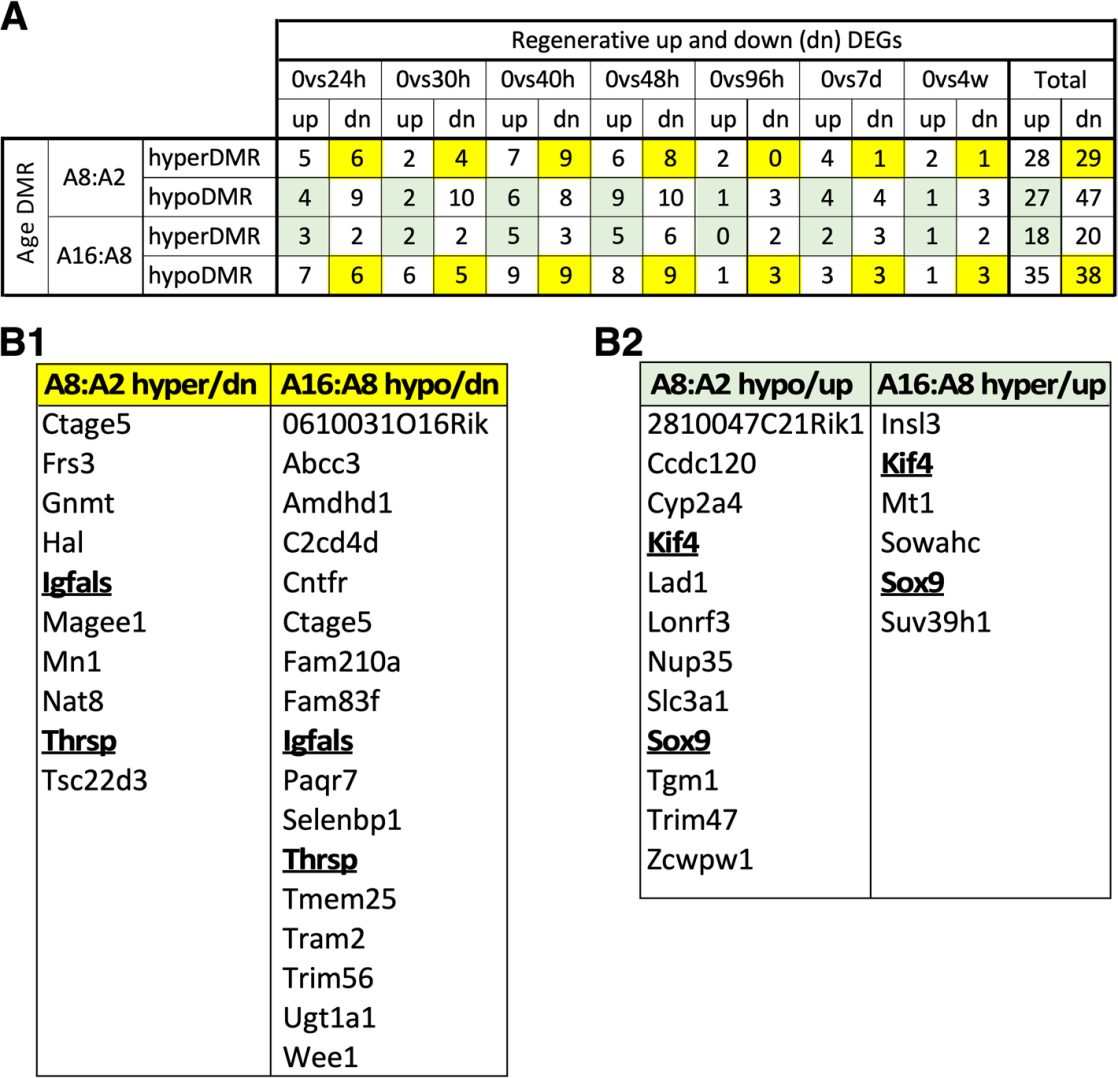
Regenerative DEGs inversely regulated by aging DMRs. **A** Numbers of regenerative up-DEGs and down-DEGs whose promoter regions were hypomethylated or hypermethylated during early (A8:A2) and mid-to-late (A16:A8) aging, respectively. Genes whose promoter regions were synchronously mapped by early aging DMRs and inversely mapped by mid-to-late aging DMRs were shaded in yellow for down-DEGs or green for up-DEGs and listed in (**B1**) and (**B2**), respectively. Genes regulated by both early and mid-to-late aging DMRs were bolded

To determine if regenerative DEGs are differentially methylated during the aging process, we mapped early (2-to-8 m/o) and mid-to-late (8-to-16 m/o) aging DMRs to the promoter (TSS-7 k + 3 k) regions of regenerative DEGs (Fig. 6A, Additional file 10: Table 8). During 2-to-8 m/o, 28 and 29 hyper-DMRs were mapped to the promoter regions of up- and down-DEGs, respectively, and 27 and 47 hypo-DMRs were mapped to the promoter regions of up and down-DEGs, respectively. During 8-to-16 m/o, 18 and 20 hyper-DMRs were mapped to the promoter regions of up- and down-DEGs, and 35 and 38 hypo-DMRs were mapped to the promoter regions of up- and down-DEGs. Given that early and mid-to-late aging DMRs were related to biologically opposite events, regenerative DEGs that were synchronously mapped by early aging DMRs (i.e., hyper-DMRs to down-DEGs and hypo-DMRs to up-DEGs) but inversely mapped by mid-to-late aging DMRs (i.e., hyper-DMRs to up-DEGs and hypo-DMRs to down-DEGs) were most likely our targets of interest for the adverse effect of aging on liver regeneration. Regenerative down-DEGs mapped by early hyper-DMRs or mid-to-late hypo-DMRs were shown in Fig. 6B1. Two of them, Igfals (insulin like growth factor binding protein acid labile subunit) and Thrsp (thyroid hormone responsive), were mapped by both early hyper-DMRs and mid-to-late hypo-DMRs. Regenerative up-DEGs mapped by early hypo-DMRs or mid-to-late hyper-DMRs were shown in Fig. 6B2. Two of them, Sox9 and Kif4 (kinesin family member 4), were mapped by both early hypo-DMRs and mid-to-late hyper-DMRs. In contrast, no regenerative down-DEGs were mapped by both early hypo-DMRs and late hyper-DMRs, and no regenerative up-DEGs were mapped by both early hyper-DRMs and late hypo-DMRs.

### Expression profiles of DMR-mapped genes during liver aging and regeneration

**Fig. 7:**
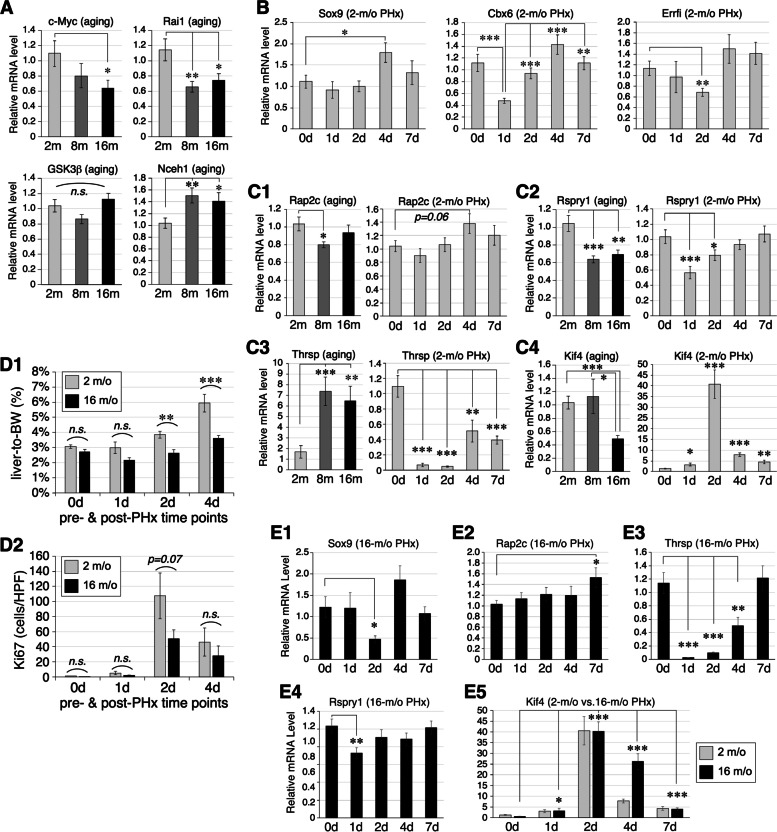
Expression profiles of DMR-mapped genes during liver aging and/or regeneration. **A** Expression of aging DMR-mapped c-Myc, Rai1, GSK3β, and Nceh1 in 2, 8, and 16-m/o livers by qRT-PCR, indicated by light grey, dark grey, and black bars, respectively. **B** Expression of regenerative DMR-mapped Cbx6, Sox9, and Errf1 in 2-m/o livers after PHx. **C** Expression profiles of two genes mapped by age-inverse regenerative DMRs (**C1**, **C2**) and two DEGs mapped by early-late aging inverse DMRs (**C3**, **C4**). **D1** Regeneration of 2-m/o and 16-m/o livers, measured by the ratio of dissected liver mass (for 0d) or remaining liver mass (for 1d, 2d, and 4d) to body weight. (**D2**) Regeneration of 2-m/o and 16-m/o livers, measured by the numbers of Ki67 + cells per high-power field (HPF = 0.126 mm.^2^). **E** Expression profiles of Sox9, Rap2c, Thrsp, Rspry1, and Kif4 in response to PHx at 16 m/o. Expression levels were normalized against that of Rplp0 as an internal reference and compared to their respective baseline (0d) or 2-m/o baseline (for E5) samples. Bars represent means (± s.e.m.) of six biological replicates with two technical repeats each (*n* = 12) in reference to the Rplp0 level of the same sample. *p < 0.05; **p < 0.01; ***p < 0.001

To validate some of the discoveries made in this study, expression profiles of lead DMR-mapped genes in liver aging and/or regeneration were measured by qRT-PCR. For the aging DMR-mapped genes listed in Fig. 2A, we confirmed that (1) c-Myc (mapped by an A16:A2 hyper-DMR) showed decreased expression in 16-m/o compared to 2-m/o livers; (2) Rai1 (mapped by an A8:A2 hyper-DMR) showed decreased expression in 8-m/o and 16-m/o livers compared to 2-m/o livers; and (3) Nceh1 (mapped a A16:A8 and A8:A2 hypo-DMR) showed increased expression in 8-m/o and 16-m/o livers compared to 2-m/o livers (Fig. 7A). GSK3β showed no difference in expression during liver aging despite that it was mapped by an A8:A2 hypo-DMR. For the regenerative DMR-mapped genes listed in Fig. 3E, we confirmed that (1) Sox9 (mapped by a R1/2/4:R0 hypo-DMR) showed increased expression in 4d compared to 0d, 1d, and 2d livers and (2) Cbx6 (mapped by a R1/2/4:R0 hyper-DMR) showed decreased expression in 1d (post-PHx) compared to 0d (pre-PHx) livers, with increasing expression on 2d, 4d, and 7d compared to 1d (Fig. 7B). In contrast, Errfi1 (mapped by a R2/4:R0 hypo-DMR) showed decreased expression in 2d compared to 0d livers. Akap5 expression showed no significant changes after PHx (data not shown). For the genes associated with mid-to-late age-inverse regenerative DMRs (see Fig. 5A2), Rap2c showed increased expression in 4d compared to 0d livers, supporting its association with a R1/4:R0 hypo-DMR (Fig. 7C1). During aging, Rap2c showed decreased expression in 8-m/o compared to 2-/mo livers, but its DMR pattern was somewhat complex, being mapped by one hyper/hypo-DMR and another hypo/hyper-DMR during early/mid-to-late aging. Rspry1 showed decreased expression in PHx 1d and 2d compared to 0d livers, consistent with its association with a R1:R0 hyper-DMR, but it also displayed decreased expression in 8-m/o and 16-m/o compared to 2-m/o livers, which was at odds with its association with an A16:A8 hypo-DMR on the same site (Fig. 7C2). For the regenerative DEGs mapped by early-late aging inverse DMRs (see Fig. 6B), Thrsp1, which was mapped by an A8:A2 hyper- and A16:A8 hypo-DMR, was significantly upregulated in 8-m/o and 16-m/o livers compared to 2-m/o livers and was significantly downregulated after PHx as early as 1d (Fig. 7C3). Kif4, which was mapped by an A8:A2 hypo- and A16:A8 hyper-DMR, was downregulated in 16-m/o livers compared to 2-m/o and 8-m/o livers and was significantly upregulated after PHx as early as 1d and most prominently on 2d (Fig. 7C4).

Next, we determined how the genes cross-regulated by aging and regeneration reacted to PHx at 16 m/o. To demonstrate that the regenerative capacity of mouse livers was indeed compromised at 16 m/o compared to 2 m/o, we measured the restored mass and mitotic activity of the remaining liver. The dissected liver mass on 0d pre-PHx and the remaining liver mass on 1d, 2d, and 4d post-PHx were measured and expressed as ratios to their respective body weights in percentages. Our results showed that the recovery of liver mass was significantly reduced in 16-m/o livers compared to 2-m/o livers on 2d (*n* = 11, 6) and 4d post-PHx (*n* = 4, 7) (Fig. 7D1). The mitotic activity, measured by the abundance of Ki67 cells per high-power field (HPF = 0.126 mm^2^), was also lower in 16-m/o livers (*n* = 7) compared to 2-m/o livers (*n* = 5) on 2d post-PHx (*p* = 0.07) (Fig. 7D2). In response to PHx, we found that aging had a noticeable effect on reducing the levels of Sox9, most significantly on 2d (Fig. 7E1), as well as delaying the upregulation of Rap2c from 4 to 7d (Fig. 7E2). Conversely, the level of Thrsp, although still suppressed during PHx-induced regeneration, was significantly elevated on 7d in 16-m/o compared to 2-m/o livers (Fig. 7E3). Although the baseline expression level of Rspry1 were reduced by ~ 40% at 16 m/o compared to 2 m/o, its PHx-induced expression changes remained the same proportionally (Fig. 7E4). Finally, the expression of Kif4 in 16-m/o livers was reduced by 60% before PHx (Fig. 7C4) but was upregulated to the same level on 1d, 2d, and 7d or even to a higher level on 4d post-PHx compared to 2-m/o livers (Fig. 7E5).

### Expression of DNA (hydroxy)methylation enzymes during liver regeneration and aging

**Fig. 8:**
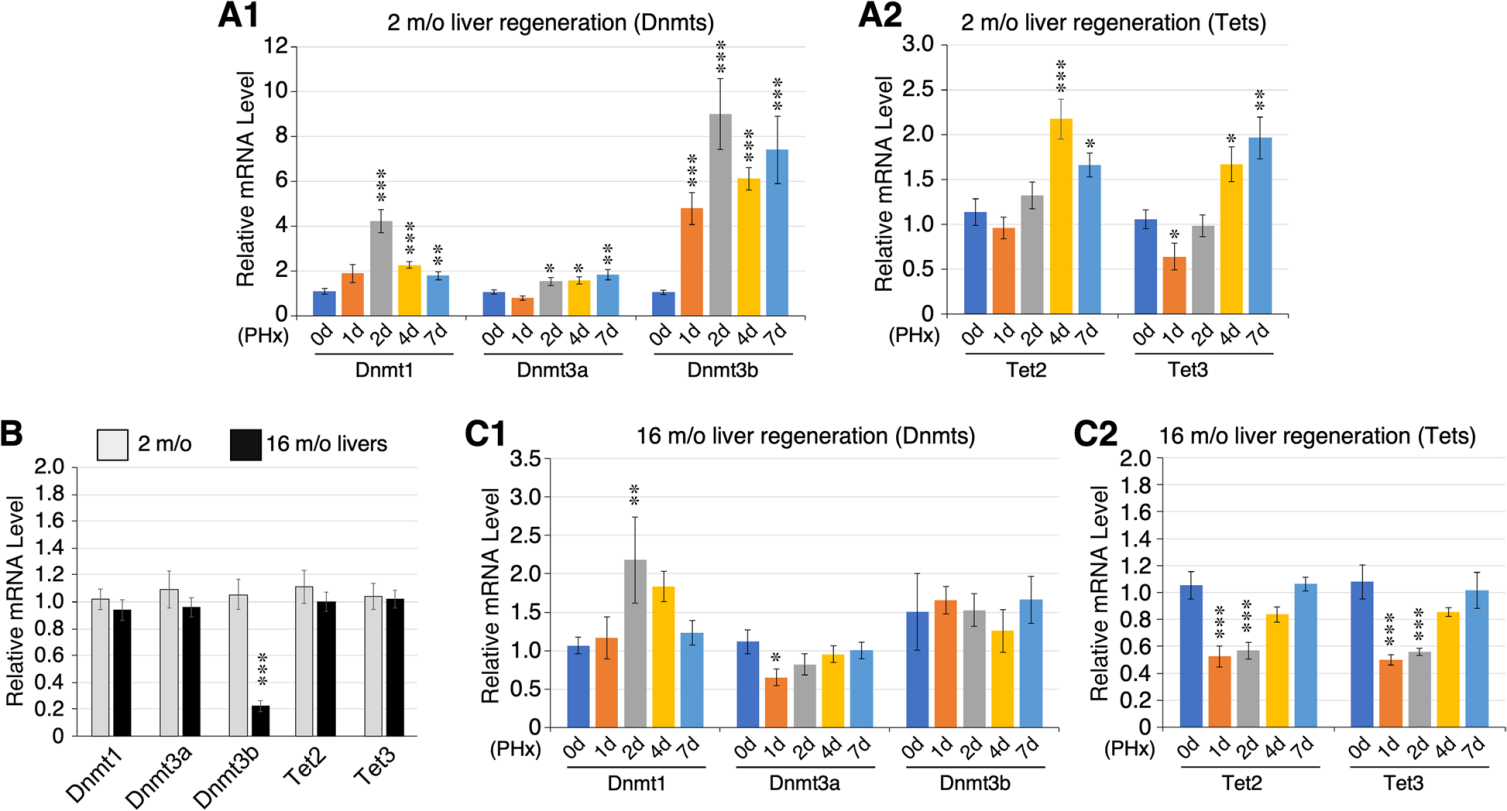
Changes in DNA (hydroxy)methylation enzyme expression during liver regeneration and aging. **A** Expression of Dnmts (**A1**) and Tets (**A2**) in 2-m/o mouse livers before (0d) and after PHx at 1d, 2d, 4d, and 7d by qRT-PCR. **B** Expression of Dnmts and Tets in 2-m/o (grey bars) and 16-m/o (black bars) livers. **C** Expression of Dnmts (**C1**) and Tets (**C2**) in 16-m/o livers before (0d) or after PHx (1d, 2d, 4d, and 7d). Expression levels were normalized against that of Rplp0 as an internal reference and compared to their respective baseline (0d) samples set arbitrarily as 100%. Bars represent means (± s.e.m.) of six biological replicates with two technical repeats (*n* = 12). *p < 0.05; **p < 0.01; ***p < 0.001

Lastly, we determined the changes in DNA (de)methylation enzyme expression during liver aging and regeneration. DNA methylation is catalyzed by one conservative DNA methyltransferase, Dnmt1, and two de novo DNA methyltransferases, Dnmt3a and Dnmt3b [49]. Conversely, active demethylation of methylated cytosine involves hydroxymethylation steps catalyzed by three Ten-eleven translocation (Tet) enzymes, Tet 1, Tet 2, and Tet 3. qRT-PCR assays were performed on 2-m/o liver samples collected before (0d) and after PHx at the 1d, 2d, 4d, and 7d time points. Our results showed that PHx-induced liver regeneration significantly increased the expression of Dnmt1 (up to fourfold) and Dnmt3b (up to ninefold) but only minimally increased Dnmt3a expression (up to 1.5-fold) (Fig. 8A1). The increase of Dnmt3b occurred shortly after PHx (1-2d) and continued for 7d, whereas the increase of Dnmt1 peaked at 2d and declined afterwards. Dnmt3a up-regulation appeared as a later and lesser event (2-7d). Compared to Dnmt1 and Dnmt3b, increased expression of Tet2 and Tet3 also occurred to a much lesser extent (~ twofold) and at later time points (4–7d) (Fig. 8A2). The level of Tet1 transcript in the liver, before and after regeneration, was too low to be reliably quantified. We next determined how aging might affect their expression in the liver. First, we compared their baseline expression levels between 2-m/o and 16-m/o livers and found that aging significantly decreases the expression of Dnmt3b and had no effect on Dnmt1, Dnmt3a, Tet2, or Tet3 (Fig. 8B). Next, we determined how aging affected the response of those enzymes to PHx-induced regeneration in 16-m/o livers. We found that aging had a noticeable effect on the PHx-induced expression of all five enzymes (Fig. 8C). Upregulation of Dnmt1 was diminished, and upregulation of Dnmt3b was completely abolished (Fig. 8C1). In 16-m/o livers, Dnmt3a, Tet2, and Tet 3 were downregulated at 1d and/or 2d after PHx and slowly returned to pre-surgical levels afterwards (Fig. 8C).

## Discussion

Liver regeneration is an age-regulated event. While DNA methylation is known to be one of the key mechanisms driving many age-associated changes, its role in liver regeneration remains to be determined. Here, we used a whole-genome single nucleotide resolution approach to identify and characterize DMRs that occurred during liver regeneration and were regulated either synchronously or inversely by the aging process. The majority of aging and regenerative DMRs were found in regions of gene structures or repeat sequences. 9.1% (169/1867) of the regenerative hypo-DMRs and 14.9% (81/548) of the regenerative hyper-DMRs were mapped to the promoter/gene regions of genes that were also differentially expressed during liver regeneration in a logically coherent way, i.e., hypo-DMRs to up-DEGs and vice versa.

### Changing landscape of DNA methylation during liver aging and regeneration

Our WGBS data show that the DNA methylation landscape in the liver changes from 2 to 8 to 16-m/o mice. Previous studies have generally established a global loss of methylation with aging in multiple tissues [33, 50, 51]. Interestingly, several recent papers have also reported no notable changes in global DNA methylation in some human cell types (e.g., brain, epidermis, muscle, heart, and liver) as well as rodent tissues (e.g., liver and hippocampus), reviewed in [31]. Now, an increasing numbers of in vitro and in vivo studies seem to support a general trend of regional differences in global DNA methylation with age, with hypomethylation on non-CpG islands and hypermethylation on CpG islands [52]. Our WGBS data show that the global methylation level in the liver barely changes, if any, from 2 m/o (74.1%) to 8 m/o (74.3%) to 16 m/o (74.6%) (Fig. 1A). Interestingly, there are more hypo-DMRs than hyper-DMRs in the A8:A2 and A16:A2 paradigms but comparable numbers of hypo-DMRs and hyper-DMRs in the A16:A8 paradigm (Fig. 1B), consistent with the idea that changes in global DNA methylation with age are region-dependent. Our findings, together with previous ones, suggest that the overall effect of aging on global DNA methylation may depend on the methods used, the tissues investigated, the age windows chosen, and the genomic regions of interest, as well as some degree of randomness. On average, ~ 1% of the CpG sites in the mouse liver genome show differential methylation changes during the aging process, whereas the other 99% of the CpG sites remain unchanged. The relatively small percentage of epigenome changed with age is not unexpected, considering that maintenance of genomic stability is crucial for the viability of cells and organisms and that major remodeling of the epigenome may result in catastrophic consequences. Those unchanged (static) methylation sites may be important for maintaining liver biology at any specific stage of age and/or regeneration but unlikely to play a direct role in driving or mediating the dynamic changes in liver regeneration with aging.

Comparing DNA methylation changes from 2-to-8 m/o to changes from 8-to-16 m/o, most liver aging DMRs occur as single events between 2 and 8 m/o (early) or 8 and 16 m/o (late). Only three DMRs show progressive changes from 2 to 8 to 16 m/o. One notable finding is the significant number of DMRs (*n* = 98) showing inverse patterns of methylation changes between 2-to-8 and 8-to-16 m/o. Analysis of their associated genes and enriched pathways reveals that early single DMRs are commonly associated with genes/pathways that promote growth and suppress cell adhesion (Fig. 2C), resembling the pattern of regenerative DMRs (Fig. 4D), whereas late single DMRs are associated with genes/pathways that promote metabolism/cell death and suppress growth. We therefore reason that aging/senescent DMRs occur primarily between 8 and 16 m/o, whereas the early aging DMRs between 2 and 8 m/o are more connected to the growth and maturation instead of degeneration of hepatocytes. Therefore, the early and late events are more likely to be biologically distinctive from or even opposite to each other, which would explain the much larger number of inverse aging DMRs compared to the number of progressive aging DMRs. In support of this model, we compared regions that are differentially methylated by aging and regeneration in the same (synchronous) or opposite (inverse) methylation direction and discovered that age-regeneration-synchronous changes occurred predominantly during the 2-to-8-m/o transition, whereas age-regeneration-inverse changes occurred predominantly in the 8-to-16-m/o transition.

In response to PHx, a total of 463 hyper-DMRs and 1648 hypo-DMRs were found in the regenerating livers from 1 to 4 days, showing a predominant pattern of hypo-DMRs. Comparing samples across different regeneration time points, hypo-DMRs were found more in the R2:R0 group than in the R1:R0 and R4:R0 groups, whereas hyper-DMRs are slightly more in the early groups (R1:R0 > R2:R0 > R4:R0). About 3.2% of the hyper-DMRs (*n* = 15) and 2.1% of the hypo-DMRs (*n* = 38) show up at all three time points. About 66.3% of the hyper-DMRs (*n* = 307) and 76.5% of the hypo-DMRs (*n* = 1,261) show up only at 1d and/or 2d. Hotspot regions for aging and regenerative DMRs include gene structures and repeated sequences. About 30–50% of the DMRs are found near the TSS, suggesting their primary functions in regulating gene expression. DMRs in regions of repetitive sequences may affect the stability of genomic structures. Some, depending on their genomic distributions, may also regulate gene expression.

### DNA methylation in regulating gene expression during liver regeneration

To explore the functional relevance of regenerative DMRs on a whole-genome scale, we extracted data on mouse DEGs in PHx-induced liver regeneration from a public RNA-seq dataset (GSE125007) [45] and identified those mapped by one or more regenerative DMRs within − 7 to + 3 kB of their TSS based on the matching pattern of hypo-DMRs to up-DEGs and hyper-DMRs to down-DEGs. Most of the DEG-mapped DMRs occur within 1d or 2d after PHx. There are more hypo-DMRs than hyper-DMRs at all time points. Pathways enriched in DMR-regulated DEGs were analyzed using GSEA in the MSigDB. DEG-mapped regenerative DMRs take place early during regeneration (1d, 2d > 4d), resulting in up-regulation of genes involved in immediate early response (e.g., protein phosphorylation, RTK signal, immune response), cell proliferation (e.g., E2F targets, KRAS signaling), and cell cycle regulation (e.g., G2M checkpoint, mitotic spindle), or, conversely, down-regulation of p53 targets, hypoxia-induced genes, and cell adhesion. Some metabolic pathways are up-regulated (e.g., glycolysis, early estrogen response), and some are down-regulated (e.g., bile acid metabolism). Several pathways are enriched in both hypo-DMR and hyper-DMR associated genes (e.g., KRAS, RNA Pol II transcription, biosynthesis, and differentiation/developmentally regulated genes), which may be due to the broad range of genes included in those gene set filters. Pathways involved in some basic cell biological functions also show up, including ribonucleotide binding, macromolecule/protein localization, and vesicle transport.

Aging affects multiple organs, including the hypothalamus-pituitary-endocrine axis in both males and females. It is therefore reasonable to assume that there will be sex-dependent as well as sex-independent DMRs. Laboratory mice, both males and females, typically reach sexual maturity at 4–7 weeks of age. The reproductive aging of female mice is characterized by progressive lengthening of estrous cycles, followed by the cessation of cycling around 12 to 15 months of age [53]. In this study, we chose to work on one sex due to the scope of whole-genome analysis. Our choice of 16-m/o female C57BL/6 mice is consistent with that of 71-w/o female C57BL/6 mice as the aged time point in the aging study by Takasugi [29]. The whole genome liver regeneration data (GSE125007) used for the screening purpose were chosen because of their liver regeneration paradigm matches ours in age, regeneration method (70% PHx), and regeneration time points, with the exception of sex. Given the possibility of sexual dimorphism, one cannot assume that all gene expression data from GSE125007 will be the same as those that might occur in female mice. Nevertheless, one can address this issue at the qRT-PCR validation step by using samples of the same age, sex, method, and time points as that of the WGBS paradigm. Among the regenerative DEGs of interest tested by qRT-PCR, some show consistent changes (e.g., Sox9, Thrsp, and Kif4) or no change (e.g., Rap2C) during regeneration as shown by the GSE125007 data. Some, on the other hand, show changes not revealed by the RNA-seq data (e.g., Cbx6, Errfi, and Rspry1). However, those unrevealed changes may also be related to the different methods used (qRT-PCR vs. RNA-seq) instead of the different sexes. Understanding how sex modifies the aging effect on liver regeneration and whether the estrous cycle plays a role in regulating liver regeneration requires further studies beyond the scope of this work.

### Age-dependent DNA methylation changes on genomic regions that are differentially methylated during liver regeneration

DNA methylation is a potential mechanism that may confer the adverse effect of aging on liver regeneration but has not yet been extensively explored. Our study provides an unbiased approach to address this issue. The numbers of DMRs that were synchronously methylated during liver aging and regeneration (*n* = 571) were significantly higher than those changed inversely (*n* = 53). The majority of the age-inverse regenerative DMRs fall within the A16:A8 window (Fig. 5A1), whereas the majority of age-synchronous regenerative DMRs fall within the A8-A2 and A16-A2 windows (Fig. 5B1). The higher number of synchronous DMRs compared to inverse DMRs may indicate that regenerative DMRs overlap more with early aging (2-to-8-m/o) than with mid-to-late aging (8-to-16-m/o) DMRs and that liver regeneration shares more common features with liver aging during 2-to-8 m/o than 8-to-16 m/o.

Of the 53 regenerative DMRs that were inversely regulated during aging, 16 DMRs were mapped to the promoter and/or gBody regions of known genes. Four of those DMR-mapped genes (i.e., Rap2c, Gata6, Duox1, and Rbbp7) are inversely regulated by mid-to-late aging DMRs and show liver-related functions. Those genes and their DMRs provide logical leads to uncover novel DNA methylation mechanisms underlying age-dependent decline in liver regeneration. We have also determined the regenerative DEGs that are synchronously regulated by early aging DMRs and/or inversely regulated by mid-to-late aging DMRs in their promoter regions. The promoter regions of two regeneration-upregulated genes, Sox9 and Kif4, are both hypomethylated from 2-to-8 m/o and hypermethylated from 8-to-16 m/o. Conversely, the promoter regions of two regeneration-downregulated genes, Igfals and Thrsp, are both hypermethylated from 2-to-8 m/o and hypomethylated from 8-to-16 m/o. Together, our data raise Rap2c, Gata6, Duox1, Rbbp7, Sox9, Kif4, Igfals, and Thrsp as potential targets where aging may inversely regulate their changed expression during liver regeneration via a promoter methylation mechanism.

### Expression profiles of DMR-mapped genes in liver aging and regeneration

Gene expression is regulated by a complex network of epigenetic (i.e., DNA methylation, histone modifications, and microRNAs) and non-epigenetic events, via *cis* elements located nearby the TSS or at a long distance away. Therefore, it should come as no surprise that only a small portion of DMRs are mapped to the vicinity of genes that show expression changes as anticipated based on their DNA methylation patterns—an observation also made in the Takasugi’s study [29]. On one hand, we have indeed validated multiple cases where DMR-associated genes exhibit corresponding changes in expression during liver aging (e.g., c-Myc, Rai1, and Nceh1, as well as Kif4 in mid-to-late aging) or regeneration (e.g., Sox9, Cbx6, Rap2c, and Rspry1). On the other hand, we have also observed DMR-associated genes that either show no change (e.g., GSK3β in liver aging and Kif4 in early aging) or display opposite changes (e.g., Errfi in liver regeneration, Rspry1 in liver aging, and Thrsp in early aging). Finally, genes not mapped by DMRs during a change of event (e.g., PHx) may still show expression changes that are regulated by either non-methylation mechanisms or by DMRs located outside our range of analysis. Examples of this include Thrsp1 and Kif4, which were not mapped by any regenerative DMR within TSS-7 k + 3 k or gBody but still were either downregulated (i.e., Thrsp1) or upregulated (i.e., Kif4) during liver regeneration, suggesting that DNA methylation may be a mechanism by which aging attenuates its expression in the liver but may not contribute to its dynamic expression during regeneration.

One keen interest of this study is to find new gene targets that are inversely regulated during liver aging and regeneration. Expression profiles of Rap2c, Thrsp, and Kif4 are congruent with this pattern. Rap2c is downregulated at 8 m/o compared to 2 m/o and upregulated in 4d PHx livers. Thrsp is significantly upregulated at 8 and 16 m/o compared to 2 m/o and also significantly downregulated in 1d, 2d, 4d, and 7d PHx livers. Kif4 is downregulated at 16 m/o compared to 2 and 8 m/o and upregulated in 1d, 2d, 4d, and 7d PHx livers. As Rap2c and Thrsp are both mapped by multiple DMRs in a complex manner and in several paradigms, it is therefore difficult to determine which DMR plays the key role in controlling their final gene expression patterns. The diverse expression profiles of DMR-mapped genes may also suggest that some targets are the causal events driving the aging process and others represent the adaptive/compensatory or integrative/phenotypic events. From a time-sequence perspective, by selecting 2, 8, and 16 m/o, we at least ensure that the DMRs identified in our search occur right before each life phase.

### Upstream events controlling DNA methylation in response to liver regeneration and aging

The causal explanation for aging remains unresolved to date. It has been proposed that aging may result from stochastic accumulation of molecular damages or follow a predetermined program. Recent studies of functional genomics reveal that age-related gene expression changes display both reversals as well as extensions of those found in development. To explain the extension, a proposed model describes that some genetic programs deployed during development may become detrimental if persistently maintained or upregulated past maturation into adulthood, possibly via epigenetic mechanisms [54]. Our study shows that, of the regenerative DMRs showing age-related changes, more are synchronously regulated than inversely regulated in their differential methylation patterns. Among those overlapping with the mid-to-late aging DMRs, 64% (44 out of 69) are inversely regulated and 36% are synchronously regulated. Our findings, although not directly addressing the causality of aging, can be consistent with the development model being one of the predetermined programs.

Some understanding on how the DNA methylation landscape is shaped during the liver regeneration and aging events may be gleaned from the expression patterns of DNA methylation enzymes (Dnmt1, Dnmt2, Dnmt3) and hydroxymethylation (Tet2, Tet3) enzymes. We found that during the regeneration of 2-m/o livers, changes in Dnmt1 and Dnmt3b expression are significant and occur early after PHx. While the increase of Dnmt1 gradually decreases after 2d, Dnmt3b remains upregulated throughout the first seven days of regeneration. Compared to Dnmt1 and Dnmt3b, the increase in Dnmt3a expression during liver regeneration is minimal and occurs late in the event. These findings suggest that both Dnmt1 and Dnmt3b are involved in hepatocyte regeneration, and that Dnmt3b is also important for newly regenerated hepatocytes to regain their differentiated features. Compared to Dnmt1 and Dnmt3b, Tet2 and Tet3 show a milder increase at later time points, which coincides with the decline in Dnmt1 expression. Upregulation of Dnmt1 and Dnmt3b at the early phase of liver regeneration is compatible with their roles in establishing and maintaining the methylation profiles of newly synthesized DNA strands in the newly born hepatocytes, whereas downregulation of Dnmt1 and upregulation of Tet2 and Tet3 at the late phase of liver regeneration may allow those partially differentiated hepatocytes to refine and regain their differentiated features. Aging decreases only the baseline expression of Dnmt3b in the liver and attenuates the response of all five enzymes to PHx-induced liver regeneration but most significantly on that of Dnmt3b. Notably, the expression profiles of Dnmt and Tet enzymes do not register exactly with the relative global abundance of hypo-DMRs and hyper-DMRs in terms of their timing during liver regeneration. This discrepancy may be explained by the time delay for Dnmt3b to reestablish the DNA methylation profiles on the newly synthesized DNA strands. Other mechanisms, such as histone modifications and gene-specific chromatin conformation changes, may also be involved.

## Conclusions

In this study, we have explored and uncovered novel DMRs and their associated genes and pathways that are inversely regulated during liver aging and regeneration to explain the adverse effect of aging on liver regeneration. These findings will be of fundamental importance in advancing our knowledge of the epigenomic changes underlying the biology of aging on liver regeneration.

## Methods

### Animal care and procedure

Animals were housed and handled in accordance with the *Guide for the Care and Use of Laboratory Animals*. All procedures were approved by the Institutional Animal Care and Use Committee (2021–0264-IBT). Seventy percent partial hepatectomy (PHx) was performed as described previously [55]. Female *C57BL/6* mice were anesthetized by isoflurane inhalation. After laparotomy, cuts were made on the falciform ligament and the membrane between the caudate and left lateral lobes of the liver. The left lateral lobe and median lobe were sequentially ligated with 5–0 silk suture and resected. The peritoneum was closed with 4–0 absorbable suture. The skin was closed using Autoclip wound clips (Becton Dickinson, #427,631).

### WGBS

Genomic DNAs were purified from fresh frozen liver tissue of four biological replicates in each group using the AllPrep DNA/RNA/Protein Mini kit (Qiagen, #80,004) and fragmented to 300–500 bp using the M220 Focused-Ultrasonicator (Covaris Inc., Woburn MA). DNA fragments were end-repaired, A-tailed, and ligated with methylated paired-end adaptors using the NEBNext Ultra™ II DNA Library Prep Kit (NEB, #E7645S/L). Adaptor-ligated DNA fragments were size-selected and bisulfite treated using the EZ DNA Methylation-Lightning™ kit (Zymo, D5030T). Library quality was analyzed using the Agilent 2100 Bioanalyzer (Agilent Technologies, G2939A) and sequenced using the Illumina NovaSeq S4 platform.

### DMR analyses

Data analyses were performed using the R version 3.4.3, Bioconductor packages, Model based Analysis of Bisulfite Sequencing data (MOABS, codes available at https://github.com/sunnyisgalaxy/moabs and https://code.google.com/archive/p/moabs/) and customized Bash scripts. The MOABS software employs a beta-binomial hierarchical model to simulate the sampling and biological variations and is capable of detecting methylation dynamics with a single-CpG resolution [39]. Adjusted credible methylation difference (*cdif*) in MOABS combines both the biological and statistical significance of differential methylation. The quality of raw WGBS data was determined using the fastqc V0.11.5 (https://www.bioinformatics.babraham.ac.uk/projects/fastqc/) and multiqc v0.9.dv0 (https://github.com/ewels/MultiQC) modules. Qualified reads were mapped to the mouse GRCm38/mm10 reference genome using the BSMAP module. Methylation calling was done using the MCALL module. DMCs and DMRs were identified using the MCOMP module, with credible methylation difference ∆β > 0.2 for hyper-DMRs or <  − 0.2 for hypo-DMRs, and *p*-value < 0.05. DMRs were annotated to the mm10 genome by functional region locations. The percentages of DMRs covered by classified genomic regions were quantified using the ChIPseeker package (https://bioconductor.org/packages/release/bioc/vignettes/ChIPseeker/inst/doc/ChIPseeker.html). The genomic distributions of DMRs were determined by downloading annotation tracks from UCSC and Refseq and performing a customized script for hotspot analysis. 1stExon (1st exon regions), 1stIntron (1st intron regions), Center30ct (from 30% left to 30% right of the center), Exon (all exonic regions), ExtGene (extended 1 kb upstream and downstream of gGene), ExtGenf (extended 1 kb upstream and downstream of gGenf), FpUTR (UTR regions close to the start site), Gene (Gene body regions), Genf (from the start of 1st exon to the start of the 2nd exon), Intron (intronic regions), Prom1k (from 1 k upstream to 1 k downstream of TSS), TSSdn1k (1 k downstream of TSS), TSSup1k (1 k upstream of TSS), TTS1k (from 1 k upstream to 1 k downstream of TTS), TTSdn1k (1 k downstream of TTS), TTSup1k (1 k upstream of TTS), TpUTR (UTR regions close to the terminate site), rmsk (all repeated regions including LINEs and SINEs), cgi (CG islands), SINE (SINE repeats), LINE (LINE repeats), LTR (LTR repeats), and Simple_repeat (short repeats). Shared DMRs between the aging and regenerative groups or between the early (2-to-8-m/o) and late (8-to-16-m/o) aging groups were defined by at least one overlapping bp, and identified using the R package. DMR-associated genes were identified by mapping DMRs to the TSS (-7 k + 3 k) and/or gene body (gBody) regions.

### Differentially expressed gene (DEG) analysis

RNA-seq raw data were extracted from the Gene Expression Omnibus (GEO) database (accession no. GSE125007) (https://www.ncbi.nlm.nih.gov/geo/query/acc.cgi?acc=GSE125007) [45]. Data quality was evaluated by fastqc and multiqc. Pair-ended reads were mapped to the mouse GRCm38/mm10 genome using Tophat V2.2.1 (https://github.com/infphilo/tophat). Uniquely mapped reads were extracted using the same tools as inputs for differential gene expression analysis. Cufflinks (http://cole-trapnell-lab.github.io/cufflinks/cuffdiff/) was utilized to assemble the transcriptome and quantify gene expression levels with reads per kb per million (FPKM). DEGs were identified using Cuffdiff V2.2.1 (http://cole-trapnell-lab.github.io/cufflinks/cuffdiff/) with corrected *p* values ≤ 0.05 and absolute fold changes ≥ 2. Heatmaps were generated using the R gplot module.

### Gene Set Enrichment Analysis (GSEA)

Pathways enriched in DMR-mapped DEGs were analyzed using the MSigDB gene sets on the UCSD/Broad Institute website (http://www.gsea-msigdb.org/gsea/msigdb/annotate.jsp). Lists of genes were run against the hallmark (H), canonical pathways (CP), and gene ontology (GO) gene sets with the false discovery rate (FDR) *q*-value set at < 0.05. Analyses of DMRs as cis-regulatory regions for pathways were performed using GO terms provided by the GREAT version 4.0.4 (http://great.stanford.edu/public/html/).

### Quantitative RT-PCR assay

Total RNA was extracted from liver tissue using the TRIzol™ reagent (Invitrogen) and reverse-transcribed into 1st-strand cDNA using the M-MLV reverse transcriptase (Promega). PCR reactions were performed using the MyiQ single-color real-time PCR detection system and supermix SYBR green reagent as described previously [56, 57]. Tm was set at 60 °C for all reactions. The ΔC(t) values were determined by comparing target messages to two reference messages, Rplp0 and Rps3. ΔΔC(t) values were calculated by comparing all samples to their respective baseline samples (set as 100%). Data represented 6 biological replicates with two technical repeats. Primer sequences are listed below.

**Table:** 

Mouse	Sense primer	Anti-sense primer
c-Myc	CAGCGACTCTGAAGAAGAGCAAG	TAGTTGTGCTGGTGAGTGGAGAC
Rai1	5′-ATGAAGGTAGCTGTGGACATGC-3′	5′-TCCTCAATGAACGTGCAACCTG-3′
GSK3β	5′-TTCTGGAGAACTGGTTGCCATC-3′	5′-GAACATAGTCCAGCACCAGGTT-3′
Nceh1	5′-GGCTAGTGCAAAGATCAGCTAC-3′	5′-TAGTGGCACGGATGACATCATG-3′
Cbx6	5′-ATTCTGGATTCGAGGCTCATCG-3′	5′-AATGCACATCACTGATGCGGAG-3′
Sox9	5′-CAGACTCACATCTCTCCTAATGC-3′	5′-CAGATCAACTTTGCCAGCTTGC-3′
Errfi1	5′-TGCTCAGGATATTCGAGTCCCA-3′	5′-CCGAGCAGCACATCCAACAGTT-3′
Rap2c	5′-TGCCTCCATGAGAGATCTGTAC-3′	5′-TAGGATGAGTGGGACTTTCTCG-3′
Rspry1	5′-GCTACTCGAGACAGCAAGTTTC-3′	5′-GTCTAACAGGAACCCTACTGTG-3′
Thrsp	5′-AAGAAGACAGGATCTCGGAGGA-3′	5′-GGTCTTCATCAGTCTTCTCTCG-3′
Kif4	5′-AGTCAAGGTGTCCAAGAAGAGC-3′	5′-GAATTGTCCTGGTTCTGATGGC-3′
Dmnt1	5′-CCACCTCGACCTGGTTTGATAC-3′	5′-CATGAATTGCTTTGGCACACCC-3′
Dmnt3a	5′-GAAGCAGACCAACATCGAATCC-3′	5′-TACCTTGGCTTTCTTCTCAGCC-3′
Dmnt3b	5′-CTCCAGCCTTCTGAATTACACGC-3′	5′-ATTGCTATGTCGGGTTCGGACAG-3′
Tet1	5′-GGAAATGCGAGGTGCTCAAAAAG-3′	5′-TTCCCCATGACCACGTCTACTG-3′
Tet2	5′-GAAACTGTTGTTGTCAGGGTGAG-3′	5′-TTGGAGCAATGACAGTAGCCAG-3′
Tet3	5′-ACCAGATCTGCAAACTCCGCAAG-3′	5′- TGAATCTCCATGGTACACTGGCC-3′
Rplp0	5′-CTGAAGTGCTCGACATCACA-3′	5′- AGTCTCCACAGACAATGCCA-3′
Rps3	5′-ATGGCGGTGCAGATTTCCAA-3′	5′-CATTCTGTGTCCTGGTGGC-3′

## Supplementary Information

**Additional file 1: Fig. S1.** Analyses of bisulfite-converted genomic DNA (gDNA) libraries (**A**) Flow chart showing major steps in whole-genome bisulfite sequencing (WGBS). (**B**) Bioanalyzer analysis of the length and integrity of four examples of bisulfite-converted libraries prepared from 2-m/o (A2R0), 8-m/o (A8R0), and 16-m/o (A16R0) baseline livers and 2-m/o regenerating livers (2d after 70% partial hepatectomy, A2R2). (**C**) Quality analysis of WGBS data of 12 methylomes of 2, 8, and 16-m/o mouse livers with four biological replicates for each group. (**D**) Quality analysis of WGBS data of 12 methylomes of 2-m/o regenerating livers at 1d, 2d, and 4d after 70% partial hepatectomy (n=4 for each group).

**Additional file 2: Fig. S2.** Distributions of aging DMRs in different genomic elements Numbers of aging DMRs found in genomic regions containing gene structures versus intergenic regions (**A**) or in repeat versus non-repeat sequences (**B**). Abbreviations: TSS-7k, within 7kB upstream of TSS; GB, gene body; IG, intergenic region; LINE, long interspersed nuclear elements; SINE, short interspersed nuclear elements; SimRep: simple repeats (micro-satellites); Satellite, satellite repeats; Others: other repeat categories as defined in RMSK from the UCSC genome browser (https://genome.ucsc.edu/cgi-bin/hgTables?db=hg38&hgta_group=rep&hgta_track=rmsk&hgta_table=rmsk&hgta_doSchema=describe+table+schema); nonRep: non-repeat regions.

**Additional file 3: Table 1.** Aging differentially methylated regions (DMRs) identified by comparing between 16-m/o (A16), 8-m/o (A8), and/or 2-m/o (A2) livers (n = 4 for each group). DNA methylation data for individual aging samples are available in GEO (GSE211999).

**Additional file 4: Table 2.** Four distinct subsets of aging DMRs based on their changing patterns from 2-to-8 m/o and from 8-to-16 m/o, including those found: (1) only in the 2-to-8-m/o paradigm (early single), (2) only in the 8-16-m/o paradigm (late single), (3) in both 2-to-8-m/o and 8-to-16-m/o paradigms in the same (progressive) direction, and (4) in both paradigms in opposite (inverse) directions. Associated genes were mapped by the DMR locations in their promoter and/or gene body regions.

**Additional file 5: Table 3.** Regenerative DMRs by comparing regenerating livers at 1d (R1), 2d (R2), and 4d (R4) after 70% partial hepatectomy (PHx) to pre-surgical livers (R0) (n = 4 for each group). DNA methylation data for individual regeneration samples are available in GEO (GSE211999).

**Additional file 6: Table 4.** Regenerative DMRs identified at single timepoints (1d, 2d, or 4d) or shared by two or more timepoints during liver regeneration induced by PHx).

**Additional file 7: Table 5.** Mapping of regenerative DMRs to the promoter regions (-7kB to 3kB of transcriptional start site) of differentially expressed genes (DEGs) during PHx-induced liver regeneration. Hypo-DMRs were mapped to up-DEGs and hyper-DMRs were mapped to down-DEGs.

**Additional file 8: Table 6.** Age-synchronous and age-inverse regenerative DMRs.

**Additional file 9: Table 7.** Mapping of age-inverse regenerative DMRs to the promoter (TSS7k3k) and gene body (gBody) regions and age-synchronous regenerative DMRs to the promoter regions (TSS7k3k) of genes.

**Additional file 10: Table 8.** Mapping of aging DMRs to the promoter regions (TSS7k3k) of regenerative DEGs in both synchronous (hyper-DMRs to down-DEGs; hypo-DMRs to up-DEGs) and inverse (hyper-DMRs to up-DEGs or hypo-DMRs to down-DEGs) patterns.

## Data Availability

All data generated or analyzed during this study are included in this published article, its supplementary information files, and publicly available repositories. Original DNA methylation data for aging and regeneration are available in GEO (GSE211999: https://www.ncbi.nlm.nih.gov/geo/query/acc.cgi?acc=GSE211999). Raw sequencing data for aging are available in SRA (BioProject No. PRJNA868694: https://www.ncbi.nlm.nih.gov/sra/?term=PRJNA868694). Processed DMR data are provided as supplementary materials.
