# Supplementary figures and images for: Epigenome-wide analysis of aging effects on liver regeneration
Source: BMC Biol. 2023 Feb 13;21:30. doi: 10.1186/s12915-023-01533-1 (PMC9926786; doi:10.1186/s12915-023-01533-1)

Figure S1

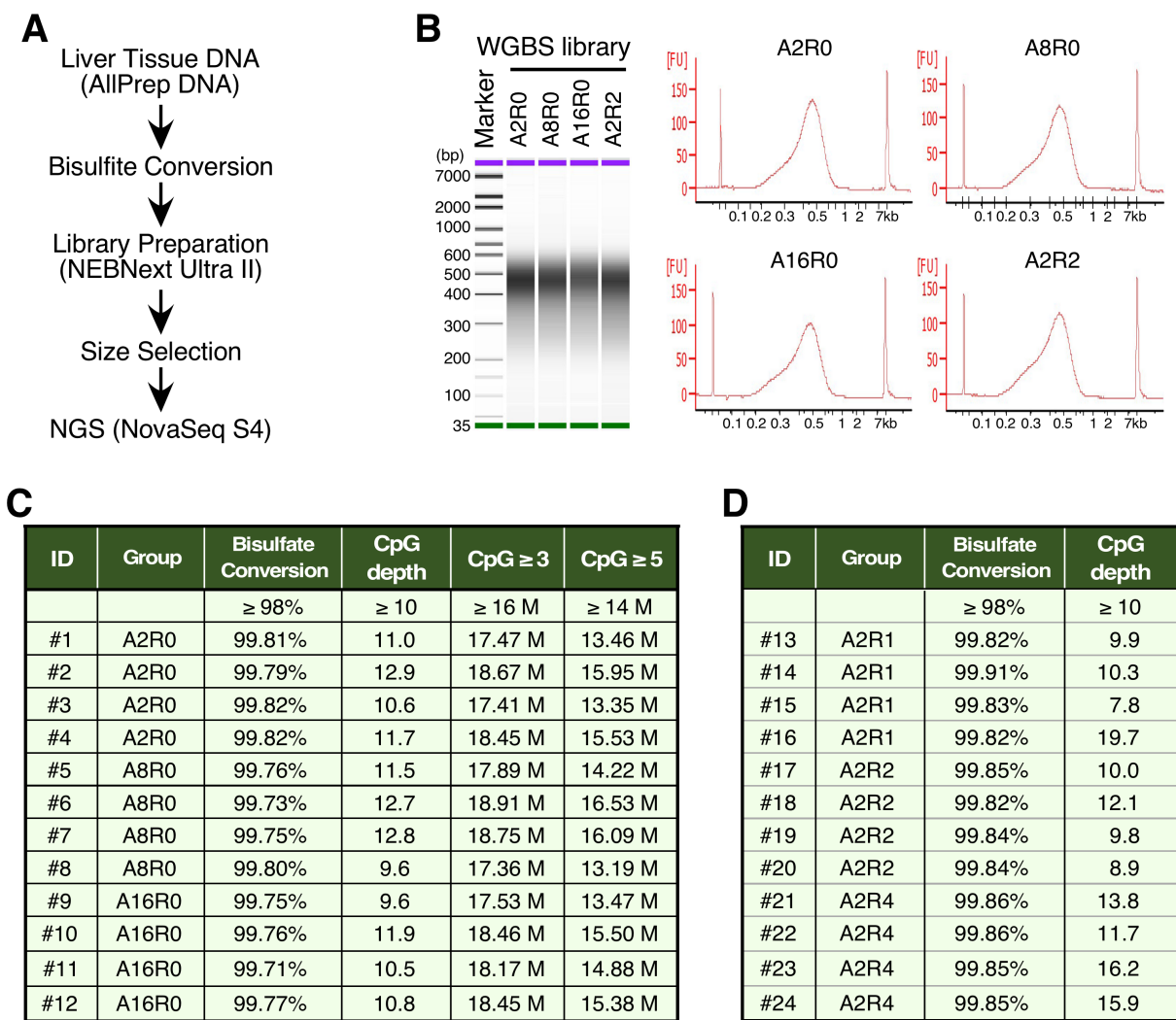

Supplement: Supplementary file 1 — Additional file 1: Fig. S1. Analyses of bisulfite-converted genomic DNA (gDNA) libraries (A) Flow chart showing major steps in whole-genome bisulfite sequencing (WGBS). (B) Bioanalyzer analysis of the length and integrity of four examples of bisulfite-converted libraries prepared from 2-m/o (A2R0), 8-m/o (A8R0), and 16-m/o (A16R0) baseline livers and 2-m/o regenerating livers (2d after 70% partial hepatectomy, A2R2). (C) Quality analysis of WGBS data of 12 methylomes of 2, 8, and 16-m/o mouse livers with four biological replicates for each group. (D) Quality analysis of WGBS data of 12 methylomes of 2-m/o regenerating livers at 1d, 2d, and 4d after 70% partial hepatectomy (n=4 for each group). [file 12915_2023_1533_MOESM1_ESM.pdf]
