# Supplementary material for: Epigenome-wide analysis of aging effects on liver regeneration
Source: BMC Biol. 2023 Feb 13;21:30. doi: 10.1186/s12915-023-01533-1 (PMC9926786; doi:10.1186/s12915-023-01533-1)
Supplement: Supplementary file 2 — Additional file 2: Fig. S2. Distributions of aging DMRs in different genomic elements Numbers of aging DMRs found in genomic regions containing gene structures versus intergenic regions (A) or in repeat versus non-repeat sequences (B). Abbreviations: TSS-7k, within 7kB upstream of TSS; GB, gene body; IG, intergenic region; LINE, long interspersed nuclear elements; SINE, short interspersed nuclear elements; SimRep: simple repeats (micro-satellites); Satellite, satellite repeats; Others: other repeat categories as defined in RMSK from the UCSC genome browser (https://genome.ucsc.edu/cgi-bin/hgTables?db=hg38&hgta_group=rep&hgta_track=rmsk&hgta_table=rmsk&hgta_doSchema=describe+table+schema); nonRep: non-repeat regions. [file 12915_2023_1533_MOESM2_ESM.pdf]

Figure S2

A

	TSS-7k	GB	IG	Total
**A8:A2**				
hyper-DMR	161	88	183	432
hypo-DMR	204	180	315	699
**A16:A2**				
hyper-DMR	222	150	269	641
hypo-DMR	359	332	582	1273
**A16:A8**				
hyper-DMR	159	138	277	574
hypo-DMR	162	129	255	546

B

	LINE	SINE	SimRep	Satellite	Others	nonRep	Total
**A8:A2**							
hyper-DMR	23	30	77	2	73	227	432
hypo-DMR	23	91	78	1	93	413	699
**A16:A2**							
hyper-DMR	31	47	120	2	108	333	641
hypo-DMR	30	170	104	3	184	782	1273
**A16:A8**							
hyper-DMR	41	69	124	4	103	233	574
hypo-DMR	21	53	83	3	99	287	546
